# Increased Cortical Activity in Binge Drinkers during Working Memory Task: A Preliminary Assessment through a Functional Magnetic Resonance Imaging Study

**DOI:** 10.1371/journal.pone.0062260

**Published:** 2013-04-25

**Authors:** Salvatore Campanella, Philippe Peigneux, Géraldine Petit, Frédéric Lallemand, Mélanie Saeremans, Xavier Noël, Thierry Metens, Mustapha Nouali, Xavier De Tiège, Philippe De Witte, Roberta Ward, Paul Verbanck

**Affiliations:** 1 Laboratoire de Psychologie Médicale et d’Addictologie, Université Libre de Bruxelles (U.L.B.) and U.L.B. Neuroscience Institute, Brussels, Belgium; 2 Neuropsychology and Functional Neuroimaging Research Unit at Centre de Recherches en Cognition et Neurosciences, Université Libre de Bruxelles (U.L.B) and U.L.B Neuroscience Institute, Brussels, Belgium; 3 Biologie du Comportement, Université Catholique de Louvain, Louvain-la-Neuve, Belgium; 4 Department of Radiology, Hôpital Erasme, Université Libre de Bruxelles (U.L.B.), Brussels, Belgium; 5 Laboratoire de Cartographie fonctionnelle du Cerveau, Université Libre de Bruxelles (U.L.B.) and ULB Neuroscience Institute, Brussels, Belgium; University of Texas at Dallas, United States of America

## Abstract

**Background:**

Cerebral dysfunction is a common feature of both chronic alcohol abusers and binge drinkers. Here, we aimed to study whether, at equated behavioral performance levels, binge drinkers exhibited increased neural activity while performing simple cognitive tasks.

**Methods:**

Thirty-two participants (16 binge drinkers and 16 matched controls) were scanned using functional magnetic resonance imaging (fMRI) while performing an n-back working memory task. In the control zero-back (N0) condition, subjects were required to press a button with the right hand when the number “2″ was displayed. In the two-back (N2) condition, subjects had to press a button when the displayed number was identical to the number shown two trials before.

**Results:**

fMRI analyses revealed higher bilateral activity in the pre-supplementary motor area in binge drinkers than matched controls, even though behavioral performances were similar. Moreover, binge drinkers showed specific positive correlations between the number of alcohol doses consumed per occasion and higher activity in the dorsomedial prefrontal cortex, as well as between the number of drinking occasions per week and higher activity in cerebellum, thalamus and insula while performing the N2 memory task.

**Conclusions:**

Binge alcohol consumption leads to possible compensatory cerebral changes in binge drinkers that facilitate normal behavioral performance. These changes in cerebral responses may be considered as vulnerability factors for developing adult substance use disorders.

## Introduction

It is well established that alcohol neurotoxicity from chronic alcohol dependence results in deleterious effects on the central nervous system, such as brain atrophy and/or dysfunction, and that these brain impairments correlate with the lifetime dose of ethanol consumed [1]. Recently, there have been several studies showing that binge drinking, which involves cycling between periods of abstinence and massive alcohol intake and affects approximately 40% of 18- to 24-year-olds in Europe [2], can lead to significant cerebral dysfunction [3], [4]. These effects are similar to those observed in chronic alcoholic patients [5]. While the definition of binge drinking stimulates debate, it is most commonly described as the consumption of five or more alcoholic drinks (four or more for women) on one occasion within a two-hour interval (according to the National Institute on Alcohol Abuse and Alcoholism), and occurring at least once in the last two weeks [6], [7] or in the last month [8], [9] with periods of abstinence between episodes. In fact, there is now mounting evidence that the practice of drinking to intoxication has become the peer norm among young people [10]. Furthermore, alcohol remains relatively inexpensive, is widely available, and is the most used mood-altering recreational drug that is employed to facilitate pleasure and the enjoyment of time out with friends [10]. Epidemiological data show that excessive use of alcohol during adolescence and young adulthood is a key factor for the development of chronic alcoholism. In both boys and girls aged 12–27 years, the prevalence alcohol use disorder (AUD), involving both abuse and dependence, is 5% with a peak incidence between ages 18 to 23 (20% in men and 10% in women) [11]. Since binge drinking and chronic alcohol-dependency induce similar brain alterations, it is important to ascertain which deficits are induced by binge alcohol consumption and which may be involved in the subsequent maintenance of alcohol use and abuse in adults. Such alcohol-induced brain alterations may also be related to difficulties in ceasing alcohol consumption, which can contribute to long-term alcohol abuse [12]. Indeed, binge drinking is considered to be an initial step towards alcohol-dependence [13]–[15].

Over the past few years, numerous studies have focused on elucidating the neural effects of binge drinking. These studies have mostly shown that while binge drinking may not induce behavioral changes that are as evident/serious as chronic alcoholism, it can provoke considerable cerebral change that is comparable to that observed in cases of alcohol dependence [16]–[26]. Indeed, electrophysiological studies have shown that binge drinking is particularly deleterious for brain functioning, rapidly leading to delayed cerebral activation throughout the information-processing stream, as reported in a nine-month test-retest study [20]. Moreover, these anomalies were not only due to the quantity of alcohol consumed, but also to the specific harmful effect of the consumption pattern (i.e., alternating between strong intoxication and withdrawal periods) [21]. This finding was not surprising, as previous animal studies have shown that repeated exposure to and withdrawal from ethanol provoked amplified neural excitability and excitotoxic cell death [27]. Furthermore, some brain imaging studies have shown that binge drinking in adolescence is associated with both decreased (occipital, hippocampal, and prefrontal areas) and increased (amygdala, insula, parietal, and superior frontal regions) brain activity during memory and decision making tasks, such as the Iowa Gambling task [23]–[26], which is sensitive for detecting decision-making impairments (i.e., how to decide advantageously in a complex situation) [28]. This led to the “compensation hypothesis” [23], [24], which suggests that binge drinkers experience a reorganization of brain functioning that involves reduced and increased activations, in order to make up for their behavioral deficits. According to a ‘functional compensation view’, decreases or absences in activation reflect deficits in brain function, and the concomitant increases in activation reflect ‘attempted’ or ‘successful’ compensation for these deficits [29]. However, it remains unclear whether these compensatory activations reflect the recruitment of different regions and processes (assuming that regional process-specificity does not change with alcohol consumption), and/or alterations in the processes mediated by the recruited regions (as a result of neural plasticity and regional changes in process-specificity due to alcohol consumption) [30].

In addition, recent functional magnetic resonance imaging (fMRI) studies have linked binge drinking with decreased performance and brain compensatory strategies [23], [26]. In contrast, others have demonstrated differences in electrophysiological components of binge drinkers compared to controls, mainly through the use of event related potentials (ERPs) and simple oddball tasks (in which participants have to detect rare target stimuli among frequent standard ones); however, they did not observe behavioral modifications [20], [22]. Therefore, it is questionable whether binge drinkers and matched controls, who *behave in the same way*, would or would not display similar brain network involvement. In other words, if no behavioral deficit is observed, will binge drinkers display a different pattern of neural activity and show similar performance level than controls? This question is highly relevant, as a positive answer would mean (i) that even in the absence of visible “behavioral” modifications, binge drinking may induce latent neural changes that could affect subsequent behavior (e.g., alcohol misuse); and (ii) that neuroimaging techniques are useful to appraise even subtle modulations of brain activity underlying minor cognitive restrictions that might be missed at the behavioral level, particularly in a population of young binge drinkers where such abnormalities are not as pronounced as in pathological populations.

In a previous study, Pfefferbaum et al. [31] compared alcoholics and controls in a visuospatial working memory n-back task, in which a volunteer is asked to monitor a series of stimuli and to respond whenever a stimulus is presented that is the same as the one presented n trials previously (where n is a pre-specified integer usually 1, 2, or 3). The task requires online monitoring, updating, and manipulation of remembered information and is therefore assumed to place great demands on a number of key processes within working memory [32]. Despite similar performance, alcoholics activated a different neural system compared to control subjects in order to function at control levels [31]. Rather than activating the dorsal “where?” neural stream and dorsolateral prefrontal cortex like the control subjects, the alcoholics activated the ventral neural “what?” stream and ventrolateral prefrontal cortex [33]. Such data are consistent with a functional reorganization of the brain systems invoked by alcoholic individuals when engaged in a spatial task requiring working memory, thereby reflecting either strategy differences in the approach taken to perform tasks, or default brain systems engaged when the optimal ones are compromised by disease or other disturbances [31].

The main aim of this present study was to compare brain activation of binge drinkers with paired controls using fMRI during completion of a two-back task. We expected that (i) by controlling for some individual as well as short-term memory factors, both groups would perform the task similarly; and that (ii) even in the absence of behavioral differences between the groups, binge drinkers would recruit a different neural network to display a performance level equal to that of controls. Thus, we could then index brain reorganization due to the neurotoxic effects of binge alcohol consumption. These data will contribute to the growing body of literature emphasizing the need for more education about the dangers of binge drinking, and for reconsideration of standard practices used to direct alcohol marketing toward young people [34].

## Materials and Methods

### Participants

First, we conducted a general screening of 150 students from the Faculty of Psychology of the University of Brussels (Belgium) in order to ascertain patterns of alcohol consumption. For this purpose, they filled in a questionnaire assessing alcohol-drug consumption characteristics as well as personal data and psychological measures. On the basis of these self-reported data, groups of participants were defined as followed. Exclusion criteria for students included major medical problems, conditions of the central nervous system (including epilepsy and history of brain injury), visual impairment, past or current drug consumption (other than alcohol, cannabis and tobacco), and alcohol abstinence. Students could be included in the study if they had very low alcohol consumption before entering college, acquired (or not) binge drinking habits after starting university, and maintained the same drinking patterns since then.

Our main objective was to select two groups of participants who only displayed differences in their alcohol binge-drinking pattern (see Table 1 for complete descriptive data). Since there is a high co-occurrence of binge drinking and substance use, such as cannabis and tobacco [35], [36], subjects currently consuming cannabis (at least once in the month before the study) were not selected. However, a similar number of nicotine users as well as those with a family history of alcoholism (FHA) [37] were included in the final groups. The Alcohol Use Disorder Identification Test (AUDIT) was used to evaluate participants in regard to hazardous drinking, harmful drinking, or alcohol dependence [38]. In line with earlier studies [20]–[22], three variables (self-reported by participants through the use of a time-line follow-back method questionnaire assessing alcohol-drug consumption characteristics) were used to determine control and binge drinking groups: mean number of drinking occasions per week (DOW: “how many times do you consume alcohol in a week in general?”), mean number of alcohol doses per drinking occasion (ADO: “how many drinks do you consume during one drinking occasion in general?”), and mean number of alcohol doses per hour (ADH: “how many drinks do you consume during one drinking occasion in a two-hours interval?”) (one dose corresponding to 10 grams of pure ethanol). According to the definition of binge drinking used in European countries, participants who drank six or more standard alcoholic drinks (10 g of alcohol) on the same occasion at a speed of at least two drinks per hour and at most two or three times per week were classified as binge drinkers. Those who drank one to 30 days a month, but never more than five standard alcoholic drinks on the same occasion and at a maximum speed of two drinks per hour, were classified as controls.

**Table 1:** The results are expressed as number, or mean ± SD.

	Controls(n = 16)	Binge drinkers(n = 16)
**Gender** (♂: ♀**)** (*χ* ^2^(1) = .000; p = 1)	7∶9	7∶9
**Tobacco (**No: Yes**)** (*χ* ^2^(1) = 1.032; p = .310)	15∶1	16∶0
**Family history of alcoholism (**No: Yes**)** (*χ* ^2^(1) = .000; p = 1)	13∶3	13∶3
**Age (year)** (t (30) = .851; N.S.)	21.6±2.6	20.9±1.8
**Level of education (years)** (t (30) = .000; N.S.)	14.4±1.2	14.4±1.9
**Right handedness (Oldfield Inventory)** (t (30) = .368; N.S.)	84.3±17.7	81.5±24.8
**AUDIT** (t (30) = −7.628)*	3.5±1.5	15.5±6.1
**Number of drinking occasions per week (DOW)** (t (30) = −5.953)*	0.5±0.4	2.7±1.4
**Number of alcohol doses per hour (ADH)** (t (30) = −3.709)**	1.5±1.2	3.4±1.7
**Number of alcohol doses per drinking occasion (ADO)** (t (30) = −5.771)*	3.7±2.6	9.2±2.7
**BDI** (t (30) = .551; N.S.)	3±3.2	2.4±2.4
**STAI Trait** (t (30) = .710; N.S.)	47.5±10.6	45.1±8.7
**STAI State** (t (30) = .973; N.S.)	50.3±10.1	47.4±6.5
**FNE** (t (30) = .782; N.S.)	14.8±7.7	12.9±5.6
**Digit Span** (t (30) = −1.692; N.S.)	6.6±1.2	7.2±0.8
**Reverse Digit Span** (t (30) = −1.519; N.S.)	4.1±0.8	5.1±1.8

AUDIT: Alcohol Use Disorder Identification Test; BDI: Beck Depression Inventory; STAI: State and Trait Anxiety Inventory; FNE: Fear of Negative Evaluation.

* Statistically significant difference between groups at p<.001.

** Statistically significant difference between groups at p = .001.

In order to ensure that any potential difference in fMRI data would be due to binge drinking and not to other variables, groups were balanced for right-handedness (assessed with the Edinburgh scale [39]), age, gender, and education level (number of years of education completed since starting primary school). Participants were also asked to fill out questionnaires assessing psychological measures: the State-Trait Anxiety Inventory (STAI) to assess state and trait anxiety [40], the Fear of Negative Evaluation (FNE) scale to assess social anxiety [41], and the Beck Depression Inventory (BDI) to assess depression [42]. Indeed, young drinkers with depression as well as general and/or social anxiety symptoms have been shown to be at increased risk of AUD during young adulthood [43], [44]. Finally, as one main hypothesis was that our two final groups would display a similar behavioral performance during the working memory two-back task (performed in the scanner), we also used digit span and backward digit span tasks as fast, reliable, and valid measures of working memory capacity [45].

Based on these criteria, 32 undergraduate students were selected for the fMRI study and classified as controls (n = 16) or binge drinkers (n = 16). We obtained informed written consent from the participants after they were fully informed about the study. The local ethics committee of the Brugmann Hospital approved the study (“Comité d’Ethique Hospitalier CE 2010/156”). The participants were instructed to abstain from consuming drugs or alcohol 24 hours before the fMRI recording, and none of them reported any binge drinking episodes in the two days prior to both assessments. Alcohol abstinence before the test was confirmed using Alco-Sensor III breath analyzers Alcometer (Alert J5®, Alcohol Countermeasure Systems Corp, 2006), and urine was tested to control for cannabis use (Tetrahydrocannabinol; Instant-View® MultiDrug Screen Urine Test; Alfa Scientific Designs, Inc.) in controls as well as in binge drinkers. Participants were paid 50 euros for their time.

### Working Memory n-back Task

**Figure 1:**
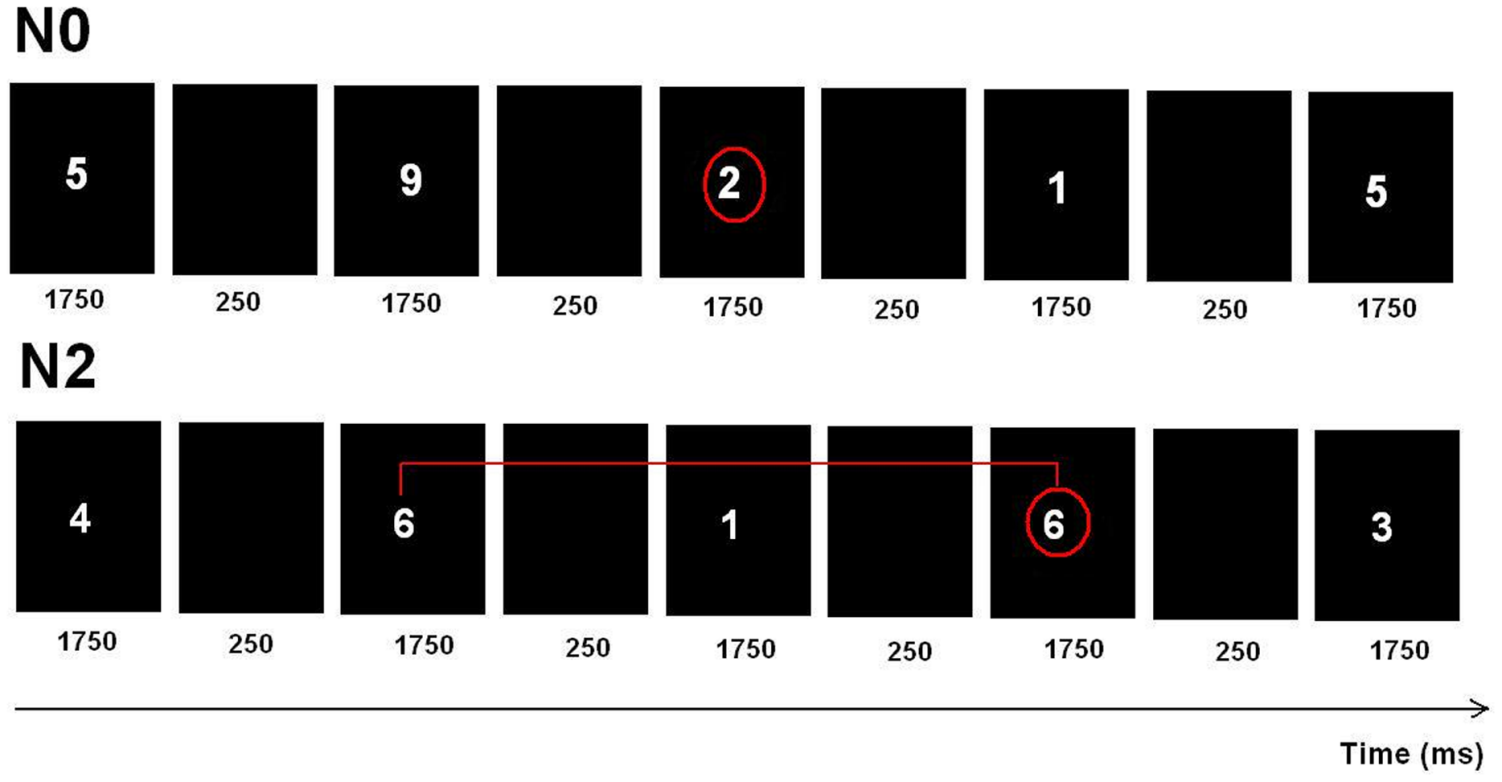
N-back working memory task. In N0 condition, participants have to detect as quickly as possible the number 2. In N2 condition, participants had to press the button when the displayed number was identical to the number displayed two trials before.

Working memory performance and underlying cerebral activity were measured using a verbal n-back task under two different conditions. In both cases, stimuli were black numbers (Arial font, size 74) displayed on a white background on the center of the screen, successively presented in pseudo-random order. In the vigilant/control zero-back (N0) condition, subjects were asked to press a button with the right hand whenever the number “2” was displayed. In the working memory two-back (N2) condition, subjects had to press the button when the displayed number was identical to the number displayed two trials before (see Fig. 1 for illustration). During the fMRI session, subjects were administered five blocks in the N0 condition alternated with five blocks in the N2 condition. Each block consisted of a sequence of 30 trials (including 10 targets) each displayed for 1750 ms with an inter-stimulus interval of 250 ms. The pseudo-random order ascertained that in N0, two targets were not successively presented, and in N2, that the same number was not repeatedly used as target (but varied randomly from 1 to 9). Each block was followed by a resting period of random duration ranging from 11 to 16 seconds, during which the instructions for the upcoming condition were displayed (i.e., either “number 2” [N0] or “same than two numbers before” [N2]). The instructions were then replaced by a fixation cross 2.5 seconds before the start of a new series of 30 numbers. All participants performed two practice blocks (one N0 and one N2) outside of the fMRI environment before scanning. During the fMRI session, stimuli were projected on a translucent screen that could be seen via a mirror fixed to the head coil and located in front of the subject, and responses were made with the right hand on a commercially available MRI-compatible keypad system (fORP; Current Design, Vancouver) connected to a PC. The timing of magnetic resonance (MR) image acquisition and stimuli presentation was synchronized using the clock signal of the MRI scanner, and all data (timing, stimuli, and responses) were recorded on the PC. Head stabilization was achieved using a head restraining foam, and MR scanner noise was attenuated using foam earplugs and headphones.

### fMRI Data Acquisition and Image Analysis

Data were acquired on a Philips Achieva 3-T (Philips Medical Systems, Best, the Netherlands) scanner using a T2* sensitive gradient echo (EPI) sequence (TR = 2130 ms; TE = 40 ms; FA 90°; SENSE acceleration factor 2.5; matrix size: 64×64×32; voxel size: 3.06×3.06×3 mm^3^). A total of 32 contiguous transverse slices were acquired, covering the whole brain. Anatomical images were obtained using a T1-weigthed sagittal 3D TFE sequence (TR = 1960 ms; TE = 4.60 ms; TI 1040 ms; flip angle 8°; FOV: 250×250 mm^2^; matrix size: 320×320×160; interpolated voxel size: 0.78×0.78×1.0 mm^3^). The MR scanner was equipped with the Quasar imaging gradients (maximum amplitude and slew rate: 30 mT/m and 200 mT/m/ms) and an 8 channel SENSE head coil.

Functional MRI data were pre-processed and analyzed with SPM8 (Wellcome Department of Cognitive Neurology, London) implemented in MATLAB 7.8 (Mathworks Inc., Sherbom, MA). The first five functional volumes in the acquisition were discarded to avoid transient spin saturation effects. Preprocessing for each individual required that functional images were (i) corrected for slice acquisition delays, (ii) realigned to the first scan of the first run (closest to the anatomical scan) to correct for within- and between-run motion, (iii) co-registered with the anatomical scan, (iv) normalized to the MNI template using an affine fourth degree ß-spline interpolation transformation and a voxel size of 2×2×2 mm^3^ after the skull and bones had been removed with a mask based on the individual anatomical images, and (v) spatially smoothed using a 8-mm full width at half maximum (FWHM) Gaussian kernel.

Data were analyzed using a mixed-effects model that aimed at showing a stereotypical effect in the population from which the subjects were drawn [46]. For each subject, a first-level intra-individual analysis aimed at modeling data to partition observed neurophysiological responses into components of interest, confounds and error, using a general linear model [47]. The regressors of interest were built using box cars positioned at each block (N2 and N0) presentation. These regressors were secondarily convolved with the canonical hemodynamic response function. Movement parameters derived from realignment of the functional volumes (translations in x, y and z directions and rotations around x, y and z axes) were included as covariates of no interest in the design matrix. High-pass filtering was implemented in the matrix design using a cut-off period of 256 seconds to remove low drift frequencies from the time series. Serial correlations were estimated with a restricted maximum likelihood (ReML) algorithm using an intrinsic autoregressive model during parameter estimation. Effect of interests were then tested by linear contrasts, generating statistical parametric maps [SPM(T)]. Here, the contrast of interest was the difference of activation between N2 and N0 conditions (N2 *vs.* N0) as the best approximation of neural activity associated with working memory. Statistical significance for images was set at p<.001 (uncorrected) and then further spatially smoothed (6 mm FWHM Gaussian kernel). They were then entered in a second-level analysis in which subjects were considered as a random effect (RFX).

At the random effect level, one-sample *t* tests were used to assess the N2 *vs.* N0 contrast in the binge and control groups separately. Two-sample *t* tests were used for a direct comparison of the N2 *vs.* N0 contrast between binge and control subjects. To test whether working memory-related changes in hemodynamic responses were associated with inter-individual variations in alcohol consumption (more so in binge drinkers than in controls), the mean number of individual ADO values were entered as a covariate of interest in the design matrix (centered within each population). Similar analyses were also performed using the DOW and ADH values in order to verify whether working memory-related changes in hemodynamic responses could also be associated with the number of weekly drinking occasions and/or number of drinks per hour, respectively. Finally, a null conjunction analysis was conducted in order to identify the brain network commonly activated in N2 *vs.* N0 for each group. ReML estimates of variance components were used to allow possible departure from the sphericity assumptions in RFX conjunction analyses [46]. Results of this conjunction analysis were considered significant at p<.05 corrected at the voxel level using Gaussian random field theory for multiple comparisons in the whole brain volume. Additionally, all results concerning group differences were only significant at an uncorrected threshold of p<.001 with a cluster extent of a least 100 voxels. These data were reported and discussed, as our two groups of participants included healthy, university, students, confronted with a simple working memory task: therefore, we did not expect great group differences, but only subtle modulations of BOLD signal. In this view, we reported False Discovery Rate (FDR) values, in order that the reader can have a precise idea about the prevalence of false positives.

## Results

### Behavioral Data

#### Accuracy rate

We conducted an analysis of variance (ANOVA; 2×2 mixed factorial design) for group (controls *vs.* bingers) as between-subject variable and task (N0 *vs.* N2) as within-subject variable. These analyses revealed an important effect of task (F(1, 30) = 22.112; p<.001), but did not disclose any significant interaction of group with task (F(1, 30) = 0.451; p = .507) or effect of group (F(1, 30) = 0.015; p = .903). Irrespective of the group, the N2 condition generated more errors than N0 (mean correct responses in N0∶99.8 [s.d.: 0.136], mean N2∶97.6 [s.d.: 0.469]). A post-hoc t-test suggested that the mean difference (2.188) was significant (p<.001) even when adjusted for multiple comparisons (Bonferroni correction). Moreover, as expected at a behavioral level, the percentage of correct responses in N2 and N0 conditions was similar between controls and binge drinkers (N2: binge drinkers *vs*. controls: t(30) = .267, p = .792; N0: binge drinkers vs. controls: t(30) = –1.379, p = .178). Accordingly, the mean number of false detections (clicking for an incorrect target in N2 condition) was similar between binge drinkers and controls (controls: 0.94 [1.18]; binge drinkers: 0.94 [1.12]).

#### Reaction time

A similar 2×2 ANOVA with group (controls *vs.* bingers) as between-subject variable and task (N0 *vs.* N2) as within-subject variable showed the main effect of task (F [1], [30] = 155.225; p<.001), while no main group effect (F[1], [30] = 0.120; p = .732) and no interaction between group and task (F[1], [30] = 2.798; p = .105) emerged. This suggested that in both groups, N0 involved faster responses than N2 (mean N0∶396 [s.d.: 9.6]; mean N2∶481 [s.d.: 11.6]). A post-hoc t-test suggested that the mean difference (85 ms) was significant (p<.001) even when adjusted for multiple comparisons (Bonferroni correction).

Overall, as anticipated, behavioral data suggested that N0 was an easier condition than N2 (involving less errors and faster correct responses), and this was true irrespective of group characteristics (see Table 2).

**Table 2:** Behavioral data (mean rate of correct response and mean correct response time ± SD) for Controls and Binge drinkers in N0 and N2 conditions.

		Controls	Binge drinkers
	**Performance**	99,6	100
**N0**		(1,08)	
	**RT**	394	398
		(44)	(64)
	**Performance**	97,75	97,5
**N2**		(2,29)	(2,46)
	**RT**	490	472
		(52)	(77)

### fMRI Data

**Figure 2:**
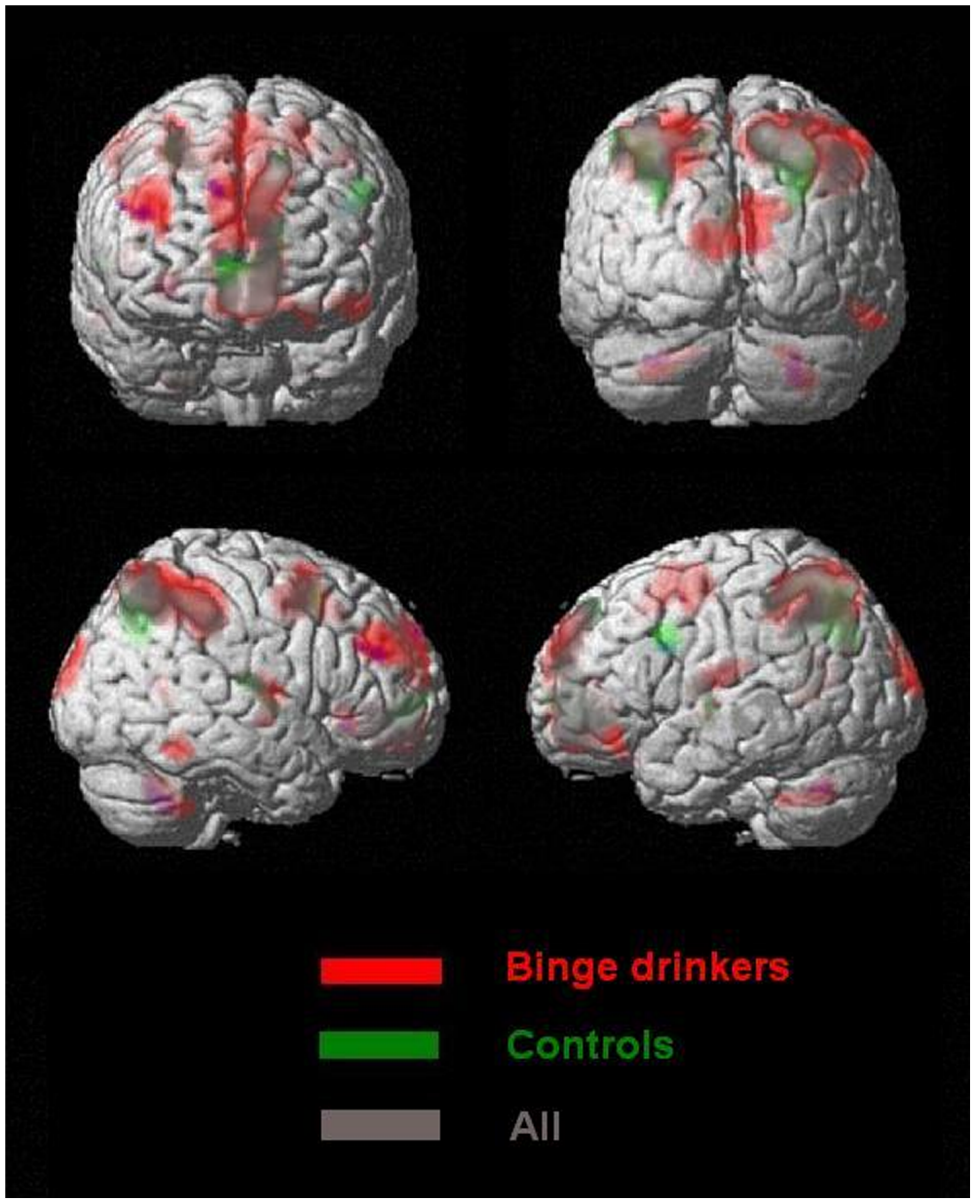
Brain areas activated during the contrast N2 vs. **N0.** Controls (Green), binge drinkers (Red) and for both groups (null conjunction analysis) (p corr = .05; cluster extent 100 voxels).

In line with previous findings in healthy adults [32], performing the working memory (N2) task, as compared to the control (N0) task, elicited increased activity in a distributed neural network mainly encompassing bilateral parietofrontal, insula and precuneus areas in both controls and binge drinkers. A conjunction analysis identified brain regions similarly involved in both populations (see Table 3 and Fig. 2).

**Table 3:** Brain areas activated during N2 vs. N0 conditions (null conjunction analysis).

MNICoordinates	Anatomical Area	K (Cluster extent)	Peak Z score
**Activations**			
26 −62 50	Right Parietal Sup	1428	6.07
4 40 −6	Right Frontal Med Orb	1846	5.90
42 −14 12	Right Insula	134	5.64
−10 −56 12	Left Precuneus	305	5.49
−42 −46 56	Left Parietal Inf	1059	5.46
24 8 46	Right Frontal Mid	230	5.36

Coordinates x, y, z (mm) are given in Montreal Neurological Institute (MNI) standard stereotactic space. All results are significant at the voxel level p<0.05 corrected, cluster extent ≥100 voxels.

**Figure 3:**
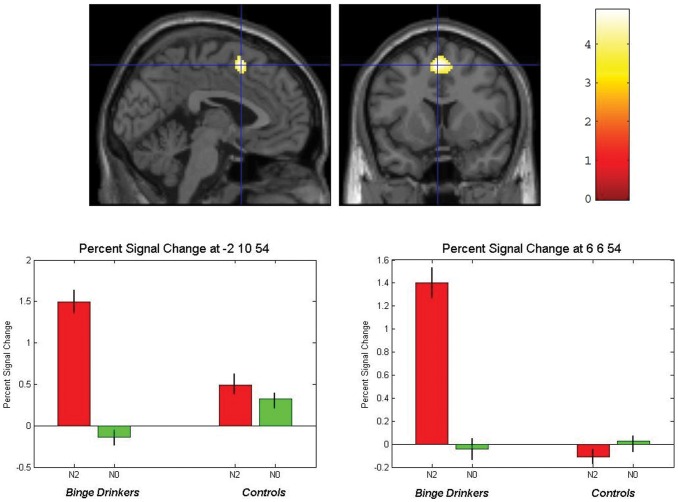
Higher working memory-related activation in bilateral pre-SMA in binge drinkers than control subjects (p uncorr <.001; cluster extent ≥100 voxels). Binge drinkers exhibited higher BOLD response in bilateral pre-SMA for N2 as compared to N0 condition, whereas controls did not.

Looking at between-population differences for the contrast N2 minus N0, our analysis disclosed an interaction effect between task condition (N2 *vs.* N0) and group (binge vs. controls) factors bilaterally in the pre-Supplementary Motor Area (pre-SMA) (*p* uncorr.001; voxels cluster extent: 251). Data analysis revealed that the interaction effect in the bilateral pre-SMA was actually due to a marked increase in blood oxygen-level dependent (BOLD) responses (as compared to baseline activity) during the N2 but not the N0 condition in binge drinkers. However, we observed that BOLD responses were similar for the N0 and N2 conditions in controls (see Table 4 and Fig. 3). The converse interaction analysis did not disclose any significant effect.

**Table 4:** Brain areas specifically activated in binge drinkers during N2 vs. N0 conditions.

MNI Coordinates	Anatomical Area	K (Cluster extent)	FDR-corr	Peak Z score
**Activations**				
−2 10 54	Left SMA	251	0.117	4.15
6 6 54	Right SMA			4.08
**Correlation with ADO values**				
0 44 38	Left Frontal Sup Medial	100	0.530	3.77
**Correlation with DOW values**				
−26 −80 −34	Left Cerebellum	432	0.178	4.75
8 −14 −8	Right Thalamus	164	0.335	4.35
14 −86 −30	Right Cerebellum	150	0.441	3.96
30 −32 20	Right Insula	115	0.441	3.90
−6 −56 −20	Left Cerebellum	148	0.751	3.64

Coordinates x, y, z (mm) are given in Montreal Neurological Institute (MNI) standard stereotactic space. All results are significant at the voxel level p<0.001 uncorrected, cluster extent ≥100 voxels. Thresholds of false discovery rate (FDR) were also reported in order that readers have a precise idea of prevalence of false positives.

**Figure 4:**
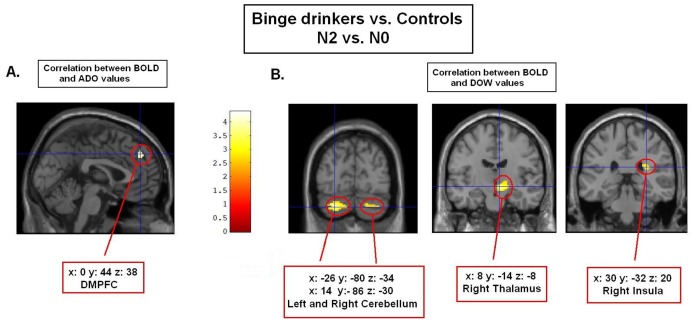
Correlation analyses. *Part A.* Higher correlation between working memory-related BOLD activity in DMPFC and ADO values in binge drinkers than control subjects (p uncorr <.001; cluster extent ≥100 voxels). The more binge drinkers ingested alcohol doses per occasion, the higher the BOLD response (reflected by level of activation change) in DMPFC was in N2 compared to N0, whereas it was not the case for controls. *Part B.* Higher correlation between working memory-related BOLD activity in cerebello-thalamo-insular regions and DOW values in binge drinkers than control subjects (p uncorr <.001; cluster extent ≥100 voxels). The higher the number of drinking occasion per week in binge drinkers, the higher the BOLD response in these regions in N2 as compared to N0, whereas it was not the case for controls.

Finally, we used ADO, ADH and DOW individual values as covariates of interest in three separate design matrices. While no significant activity emerged when ADH values were used, we found a differential relationship between ADO or DOW values and working memory-related activity (N2 *vs.* N0) in the binge *vs.* control populations. This activity was disclosed in the dorsomedial prefrontal cortex (DMPFC) when ADO values were used, whereas a joined activity in bilateral cerebellum, right thalamus, and right insula was found when DOW values were entered (*p* uncorr.001; voxels cluster extent: 100). In other words, unlike controls, there was a positive correlation in binge drinkers between (i) the number of drinks per occasion and DMPFC BOLD activity in N2 *vs.* N0 condition; and (ii) the number of drinking occasions per week and cerebello-thalamo-insular activity in N2 *vs.* N0 condition (see Table 4 and Fig. 4). The converse interaction analyses were not significant.

## Discussion

In the present study we found that even if binge drinkers and matched controls displayed a similar performance level in a working memory task, binge drinkers showed (i) higher bilateral activity in the pre-SMA; (ii) a positive correlation between the number of alcohol doses consumed per occasion and higher activity in the DMPFC region to execute the working memory condition (N2 *vs.* N0); and (iii) a positive correlation between the number of drinking occasions per week and higher activity in cerebello-thalamo-insular regions to perform in the working memory condition (N2 *vs.* N0).

Participants were challenged to an n-back working memory paradigm, contrasting N2 and N0 conditions. This task requires online monitoring, updating, and manipulation of remembered information and is therefore assumed to place great demands on a number of key processes within working memory [32]. In agreement with previous studies, a conjunction analysis showed that frontoparietal areas as well as insula and precuneus were activated in both groups. The importance of frontal and parietal regions in working memory is largely undisputed. On the one hand, frontal regions have been implicated in numerous cognitive functions that are relevant to the n-back task, including monitoring and manipulation within working memory [48], holding non-spatial information on-line [49], the specification of retrieval cues [50], or “elaboration encoding” of information into episodic memory [51]. On the other hand, parietal cortex is considered to be involved in the implementation of stimulus response mapping [52] and in the storage of working memory contents [53] as a kind of “buffer for perceptual attributes” [54]. Also, the activation of precuneus during the visual working memory task is consistent with a recollection process aided by visual imagery [55], while insula activation is considered to be a part of the inferior fronto-parietal network, which responds to behaviorally relevant rather than to expected stimuli [56]. This suggests an abstract role in extracting and processing task-relevant and salient information [57].

Analysis of the working memory tasks in binge and control subjects revealed that binge drinkers displayed increased BOLD responses in the pre-SMA (bilaterally) during the N2 condition but not the N0 task, while the controls did not. In other words, our data showed that greater activation was observed bilaterally in the pre-SMA in binge drinkers, while they displayed similar performances to controls. The pre-SMA is functionally known to be associated with more complex and cognitive controls when compared with the SMA [58]. It has been suggested that activity in this region is related to the maintenance of visuospatial attention during working memory, a process that is likely to be particularly important where delays are imposed between a stimulus and a response to that stimulus [59]. Such delays are, by definition, characteristic of the n-back tasks whereby a response is determined not by the presence of a particular stimulus alone, but by the presence of a stimulus that is identical in some predefined respect to one that has been presented n trials previously [32]. Moreover, we found that the higher the number of alcohol doses per occasion, the more the DMPFC was activated in binge drinkers in the working-memory condition. These data suggest that participants who are accustomed to higher alcohol doses have to activate the DMPFC more to continue performing the task (i.e., in a brain region that seems to be crucial in attended stimulus perception [60] and is well-known to be altered in chronic alcoholics [61]). Also, we found that the more drinking occasions per week, the more we observed conjoint activation of portions of the thalamus, cerebellum and insula in binge drinkers during the working-memory condition. These data suggest that binge drinkers who consume alcohol more times per week have to strongly activate the cerebello-thalamo-insular regions (i.e., a set of brain regions well-known to be activated by covert shifts of attention, as the n-back task required subjects to continuously shift their focus from an external to an internal frame of reference in order to compare the identity of stimuli in working memory buffers to those presented externally) [32], [62]. Among these regions, it is worthwhile to note that the insula is seen as a key anatomical target for intervention to treat addiction, as this region is thought to integrate interoceptive states into conscious feelings (e.g., craving) and into decision-making processes that are involved in uncertain risk and reward [63]. Thus, the study of this region in relation to the transition from controlled excessive consumption to alcohol dependence is highly relevant. Therefore, further experiments tagging the functioning of insula in binge drinkers may be useful to investigate whether, as compared to light drinkers, goal-directed decisions are hijacked by the activity of an impulsive system that intensifies motivation and weakens control on behavior.

Besides the potential concern linked to the fact that we have to rely on self-reported data to characterize the pattern of alcohol consumption of our participants, we are aware that a main problem of the present study is that we only reported group differences by using an uncorrected threshold (P<.001) with a minimum voxel clustering value of 100 voxels. Indeed, if, for a few datasets, this threshold may strike an appropriate balance between sensitivity and specificity, for others it cannot be appropriate, inducing therefore very different probabilities of false positives [64]. In the present study, we used two groups of healthy, university students, confronted with a simple cognitive task: therefore, we expected that no strong differences, but only subtle modulations, could emerge. However, as false positives are difficult to refute once established in the literature, this danger should be minimized. In this view, as suggested by Bennett and colleagues [64], we presented in Table 4 False Discovery Rate (FDR) values, in order that the reader can have a precise idea about the prevalence of false positives. With this mind, the present set of data should clearly be considered as preliminary. Nevertheless, besides the need for an independent replication of these results, we are convinced that the present data deserved attention. Young drinkers are often confused about what constitutes “an acceptable moderate consumption” and what damage they may actually be doing to their general health and/or brain. This may be due in part to ambiguous messages about potential positive medicinal effects of moderate alcohol consumption (e.g., reduced risk of heart attack; [65]), and also to the fact that, up to now, alterations and behavioral deficits due to alcohol consumption have only been described after long term binge drinking [66]. Our results suggest that in a very simple working memory task and in the absence of behavioral effect, binge drinking leads to brain modifications. We observed that binge drinkers recruited more neural activity at matched control performance levels. This supports earlier data suggesting that during more complex cognitive tasks, when a behavioral deficit is observable, brain “compensation strategies” might be engaged, consisting of concomitant reduction and increase of activity in distinct brain areas [23], [26]. However, we are totally aware that (1) in the present study, we did not find any relationship in binge drinkers between BOLD activity and working memory performance, so that any causal link between increased activity and performance can be drawn; and (2) it is not possible from the present study to completely discount the possibility that the differential effects observed for binge drinkers are pre-morbid in nature, i.e., existed prior to any alcohol consumption. In this view, further longitudinal studies should be designed in order to verify whether the emergence of brain differences in bingers followed (or not) the onset of drinking habits.

Also, even though positive correlations in specific brain regions were observed in binge drinkers between ADO/DOW values and the working memory condition (N2), the present data do not allow us to know whether the reported effects are *caused* by alcohol intoxication, or by the pattern of binge drinking *per se* (consisting in periods of intoxication followed by periods of abstinence), or both. Further studies should investigate this issue. For instance, a comparison of neural activity in young drinkers who consume the same amount of alcohol per week (for example, 28 doses), but with one group consuming these drinks in a regular way (four drinks each day), and the other group displaying a binge drinking pattern of consumption (14 doses in two different days of the week). In regard to our findings, it seems important to modify current information and prevention programs in order to impart the message that binge drinking is not just trivial social fun, but if continued, might favor the onset of cerebral disturbances. Moreover, these alterations in brain function can even occur *at a stage at which behavioral manifestations are not yet observable* and may lead to alcohol dependence later in life. Indeed, several promising studies have recently discovered that enhanced drug cue-reactivity or cognitive processing of substance cues can be deliberately controlled or reduced [67]. Finally, future studies on larger populations are needed to confirm our results, and longitudinal studies should be designed to explore the persistence of these brain changes when binge-drinking habits cease.
